# First-principles study of the electronic and optical properties of a new metallic MoAlB

**DOI:** 10.1038/srep39790

**Published:** 2016-12-22

**Authors:** Xiaohong Li, Hongling Cui, Ruizhou Zhang

**Affiliations:** 1 College of Physics and Engineering, Henan University of Science and Technology, Luoyang 471023, China; 2 Department of Chemistry and Center for Molecular Simulation, University of Calgary, Calgary, Alberta, T2N1N4 Canada

## Abstract

The structural, elastic, electronic and optical properties of MoAlB were investigated by first-principles calculations. The hardness of MoAlB is 12.71 GPa, which is relatively softer and easily machinable compared to the other borides. The analysis of the band structure and density (DOS) of states indicates that MoAlB has a metallic nature. The analysis of the electron localization function (ELF) shows that the Mo-B bond is a polar covalent bond with a short distance, which may increase the stability of the compound. The calculation of the phonon frequencies confirms the dynamical stability of MoAlB. Optical properties of MoAlB are investigated. In the energy range up to ~19 eV, MoAlB possesses high reflectivity and has the strongest absorption in the energy range of 0–23.0 eV. In addition, the plasma frequency of MoAlB is 20.4 eV and MoAlB can change from a metallic to a dielectric response if the incident light has a frequency greater than 20.4 eV.

“MAX-phase” is a family of layered transitional metal carbides and nitrides with general formula M_n+1_AX_n_ with n = 1–3, where M is an early transition metal, A is a group IIIA-IVA element, and X is either carbon or nitrogen^1^. Most of these phases were discovered in the 1960 s. Since the 1960 s, research on these phases has increased dramatically, and 413 subgroup and several new MAX phases were discovered^2^,^3^. Research shows that the MAX-phases possess attractive properties, combining the merits of metals and ceramics such as high melting temperature, high elastic stiffness, good machinability, and high thermal and electrical conductivity^4^,^5^,^6^. These important metallic and ceramic properties of the MAX phases are determined by their structures, which consist of stacked layers of M-X octahedra separated by mono-atomic A “metallic” layers^7^.

Mo-base MAX phases have many attractive properties and have established the research field in recent years. In 1942, Halla and Thury first described MoAlB^8^, then in 1966, Jeitschko *et al*.^9^ discovered the MoAlB (space *Cmcm*) ternary transition metal boride and found that its structure was similar to the MAX phases. In 1995, Yu and Lundstrom^10^ presented crystal growth and structure refinement of Mo_1−x_Cr_x_AlB (x = 0,31). Rieger *et al*.^11^ discovered that MoAlB has relatively lower hardness than WAlB and higher electrical conductivity than WAlB. Compared with Ti_2_AlC and Cr_2_AlC^12^,^13^, the Al content in MoAlB would form upon heating in air.

The all-electron projector augmented wave (PAW) method is an efficient method to be used in *ab initio* electronic structure calculations of periodic systems. Zhang *et al*.^14^ predicted the structure of ZrB_4_ and investigated the mechanical, and electronic properties of ZrB_4_ by using the PAW method. Wang *et al*.^15^ used the PAW method to investigate the novel superhard B-C-O phases and thought that B_4_CO_4_ is potentially superhard. Tang *et al*.^16^ investigated the phonon dispersion and elastic constants of orthorhombic CN and thought that CN is a potential superhard material, using the PAW method.

In this article, the equilibrium atomic structures of MoAlB are calculated and compared with the available experimental values by using the PAW method. Optical properties such as the dielectric function and the refractivity and electronic properties such as the density of states (DOS), the electron localization function (ELF) and the band structure were further investigated. We consider our work to be a starting point for further theoretical and experimental work for MoAlB.

## Results and Discussion

**Figure 1:**
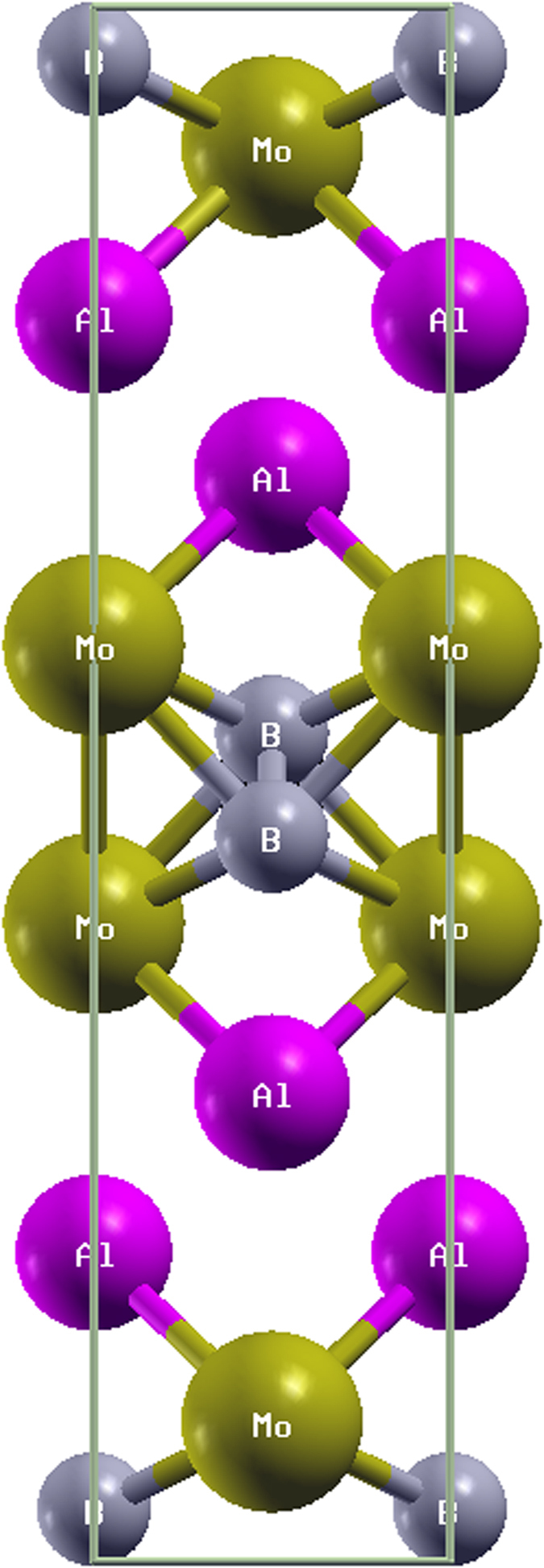
Crystal structures of the *Cmcm*-MoAlB viewed on the (001) plane.

In the present work, MoAlB is in orthorhombic *Cmcm* structure (Fig. 1), which contains four MoAlB formula units in the unit cell. The calculated and experimental values for lattice constants and atomic positions^10^,^17^ are listed in Table 1. Clearly, the calculated lattice constants are consistent with the experimental values. The lattice parameters for a, b, c are 3.1898 Å, 13.9024 Å, 3.0982 Å, respectively, with errors of 0.27%, 0.13% and 0.14% with respect to the experimental results^10^.

The bulk modulus B and shear modulus G are two important parameters in mechanics. The bulk modulus is a physical quantity that allows one to estimate rigidity, while the shear modulus is used to determine the ductility of a material. Using the CASTEP program^18^, all the elastic stiffness constants *C*_*ij*_ for the MoAlB crystal are calculated. According to the Voigt-Reuss-Hill approximation^19^, the bulk modulus B and shear modulus G can be obtained by taking the average of Voigt’s and Reuss’s schemes, which represent upper and lower bounds:


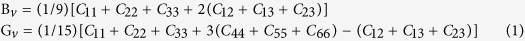



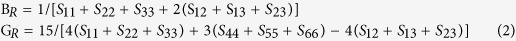


In which *S*_*ij*_ represents the elastic compliance constants consisting of the compliance tensor. Young’s modulus E and Poisson’s ratio ν can be obtained by the following equations:


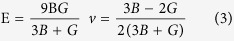


In addition, an empirical model^20^ correlating the elastic moduli and Vickers hardness was used to calculate Vickers hardness:


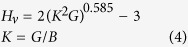


The obtained bulk modulus B, shear modulus G, Vickers hardness H_ν_, Young’s modulus E and Poisson’s ratio ν are listed in Table 1. The available experimental values are also included. Ade *et al*.^21^ synthesized MoAlB crystals and obtained a hardness of 13.6 GPa. They found that the hardness of different crystal planes ranged from 11.4 to 13.6 GPa. Sankalp *et al*.^17^ measured the hardness of the MoAlB crystal to be 10.3 GPa. In this paper, the obtained hardness is 12.71 GPa, which is closer to the experimental results^21^,^17^. In addition, compared to the other borides such as MoB (*H*_*v*_ = 23 GB)^22^, MoB_2_(*H*_*v*_ = 21–27 GPa)^23^, MoAlB is relatively softer, so it is most readily machinable and it is damage tolerant. Compared with MoB, the major difference in behavior for MoAlB is due to the mobile dislocations in the crystal structure. The bulk modulus of MoAlB is 210.99 GPa, which is smaller than that of MoB_2_ (304 GPa)^23^, but much bigger than those of TaS_2_ (155.74 GPa) and TaSe_2_ (148.39 GPa)^24^.

**Figure 2:**
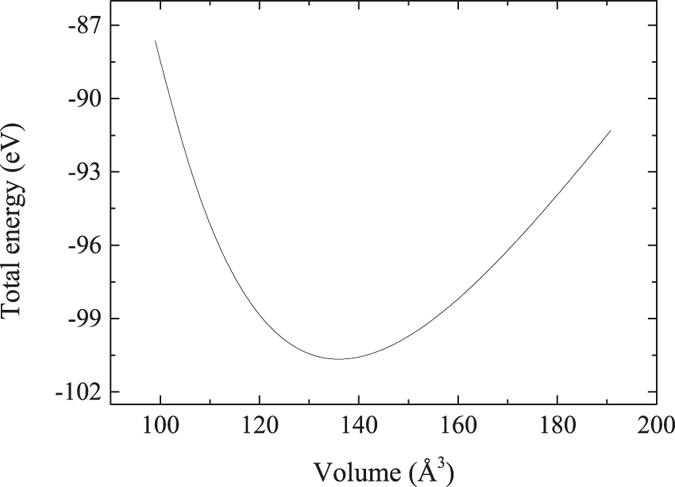
Energy versus lattice volume of MoAlB.

In this paper, we performed the minimization of the total energy for different lattice volumes at a given energy cutoff value (850 eV). Then, fitting the data of total energy and the lattice volume to the Birch-Murnaghan equation of state^25^, we obtained the bulk modulus B and the equilibrium volume V, which can be compared with the results obtained by the CASTEP calculation. The obtained bulk modulus B and equilibrium volume from the Birch-Murnaghan equation of state are 215.25 GPa and 137.21 Å^3^, respectively. Obviously, the obtained modulus B (215.25 GPa) from the Birch-Murnaghan equation of state is closer to the calculated value 210.99 GPa, and the equilibrium volume (137.21 Å^3^) is much closer to the calculated value 136.84 Å^3^ and the experimental value 137.77 Å^3^, which shows that our calculation is reliable. Figure 2 presents a plot of the energy versus lattice volume for MoAlB.

**Figure 3:**
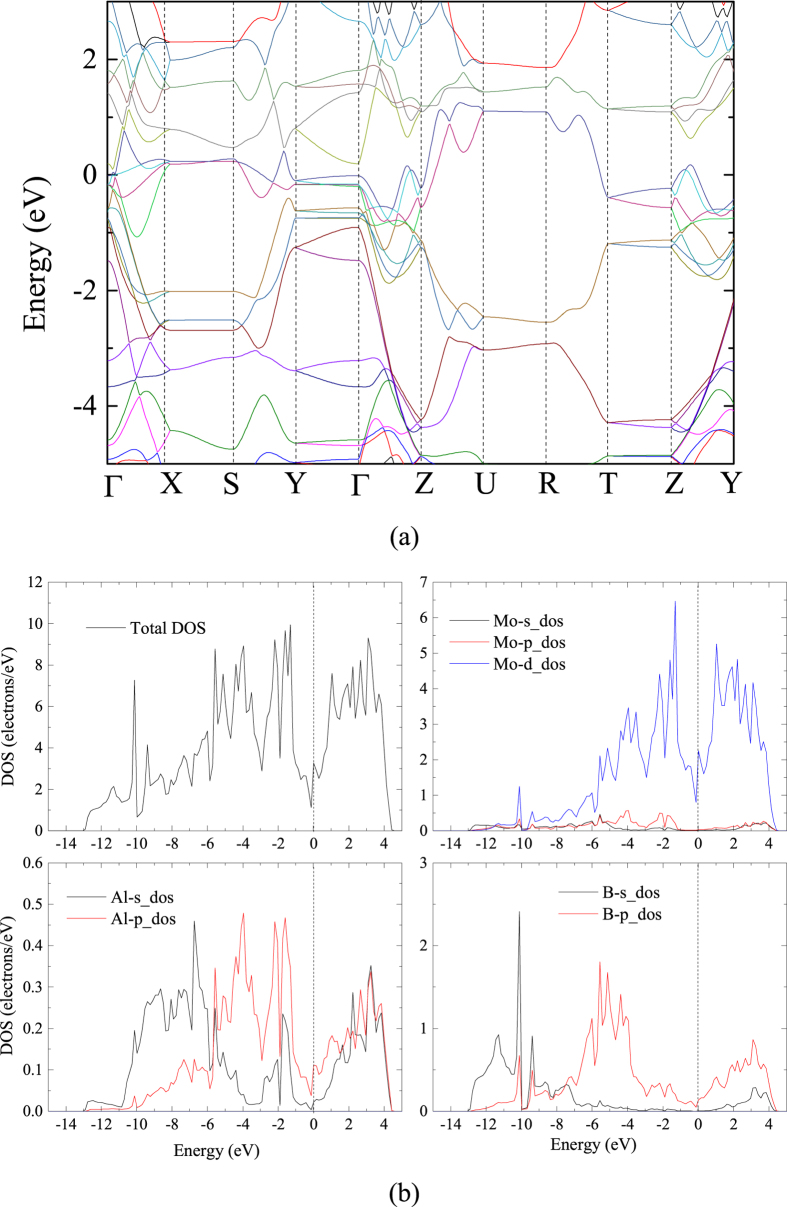
Electronic density of states and band structure of MoAlB at ambient pressure. (**a**) Band structure diagram; (**b**) Total/projected density of states. The zero of energy is at the Fermi level.

Electronic properties can be used to determine the metallic, semiconducting or insulating character of a compound. An energy gap between the valence and conduction bands can be deduced from the density of state (DOS) and band structure, which are investigated and shown in Fig. 3. The band structure calculations for the MoAlB crystal are performed along high symmetry directions of the Brillouin zone. The calculated energy band structures in Fig. 3(a) indicates clearly that the energy band curves pass through the Fermi energy, which shows that MoAlB is a metal at ambient pressure. The bands which cross E_F_ originate primarily from Mo-d states, with some contributions from B-p states. The DOS of materials can provide more information about the electronic structure. From Fig. 3(b), the relatively low DOS at the Fermi energy also reveals the metallic character of the MoAlB crystal. In addition, it also indicates that MoAlB should be stable within the perspective of electronic structure according to the free-electron model^26^.

The energy of MoAlB in the DOS ranges from −37 to 5 eV, with a dominant structure having a split peak appearing at about −36 eV. The narrow subband at about −36 eV is completely attributed to Mo-p orbitals and is well separated from other orbitals. In order to present the DOS more clearly, in Fig. 3(b), we only plotted the DOS range from −15 to 5 eV. Research shows that most of the states at the Fermi energy in MAX phases come from the d orbitals of the transition metal M^27^,^28^. This is also the case for MoAlB. The electronic states near the Fermi level are mainly contributed by the d orbital electrons of transition metal atom Mo, with some of the p orbital electrons of the B atom. The contributions from Al are nearly negligible. From −7 eV to −4 eV and 0 eV to 5 eV, the partial DOS profiles of MoAlB for Mo-d and B-p orbitals are very similar and overlap each other, which shows the strong hybridization between the orbitals of Mo-d and B-p states and confirms the strong covalent Mo-B bond.

**Figure 4:**
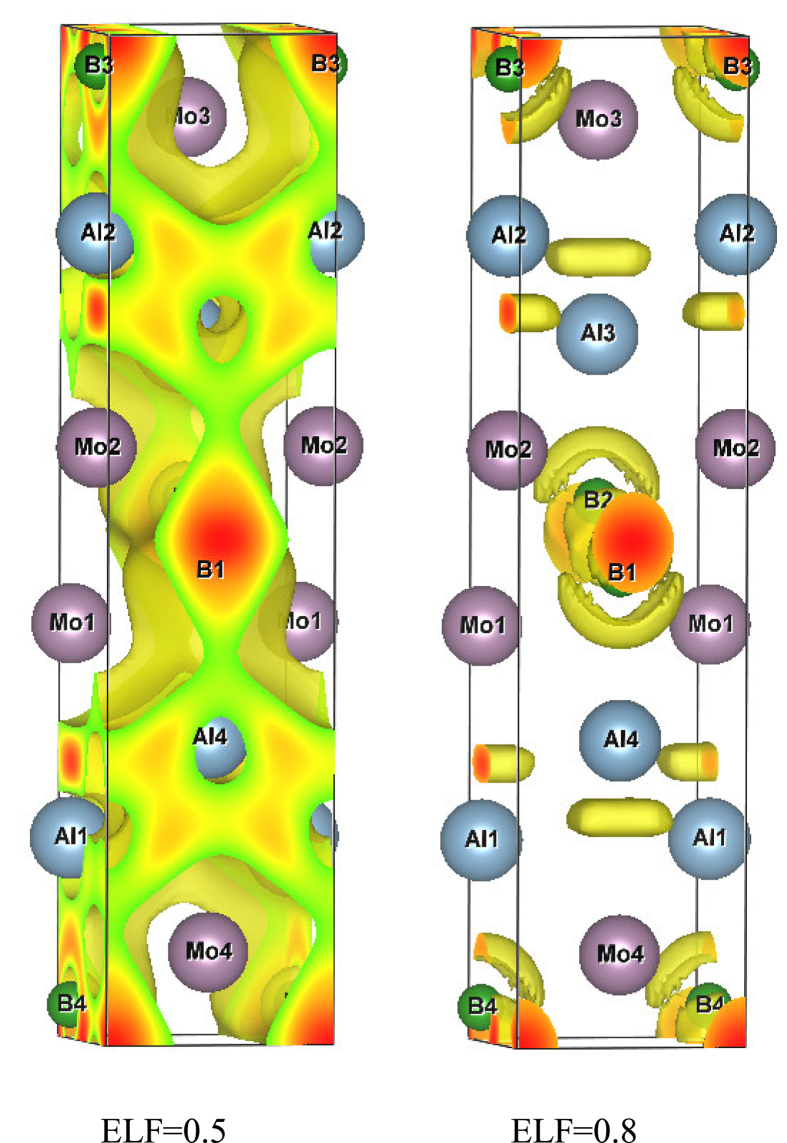
The electron localization function (ELF) of MoAlB at 0.5 and 0.8, respectively.

In order to obtain the detailed bonding character of MoAlB, the electronic localization function (ELF) was calculated. Based on the Hartree-Fock pair probability of parallel spin electrons, ELF can be used to describe and visualize chemical bonding in molecules and solids^29^. ELF ranges from 0 to 1. ELF = 1 means the perfect localization characteristic of covalent bonds or lone pairs, while ELF = 0.5 the electron-gas like pair probability (i.e., a metallic bond), ELF = 0 corresponds to no localization (or delocalized electrons). Figure 4 shows the obtained ELF of MoAlB at ELF = 0.5 and 0.8, respectively. Clearly, when ELF is equal to 0.8, ELF at the Mo sites is negligible, while it has the local maximum values at the B sites. This shows that the bond between B and Mo atoms is partially Mo-B covalent and has an ionicity with B withdrawing charge from Mo. The polar covalent bonds and the short distance (about 2.37 Å) for Mo-B bond may increase the stability of the compound. The maximum ELFs between Mo and Al atoms are 0.5. So the bonds between Mo and Al atoms are metallic.

**Figure 5:**
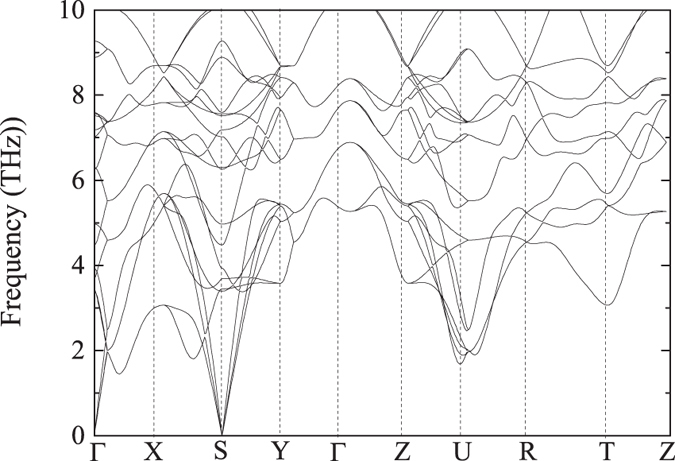
Phonon dispersion curves of the *Cmcm*-MoAlB crystal at standard pressure.

In order to determine the dynamical stabilities of MoAlB, the phonon dispersion curve is presented in Fig. 5. The absence of negative frequencies (imaginary frequencies) in the whole Brillouin zone confirms the dynamical stability of the *Cmcm*-MoAlB crystal at standard pressure.

**Figure 6:**
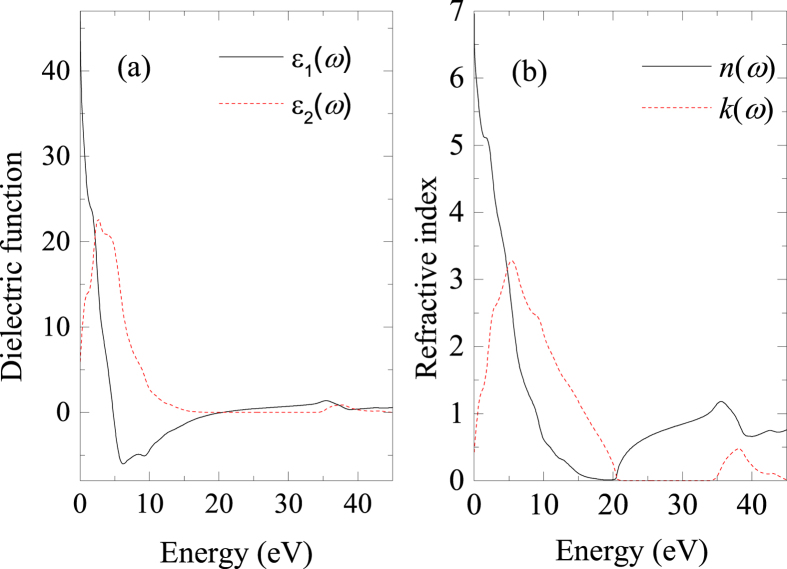
Optical properties of MoAlB. (**a**) Real part ε_1_of dielectric function and Imaginary part ε_2_ of the dielectric function; (**b**) Real part *n* of Refractive index; (**f**) Imaginary part *k* of refractive index.

Optical properties are some of the most important properties for a material, indicating a material’s response to electromagnetic radiation and, in particular, to visible light. The frequency-dependent dielectric function is an important optical parameter, 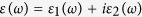, and has a close relationship with the electronic structure. The imaginary part *ε*_2_(*ω*) of the dielectric function can be expressed as:


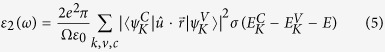


where *ω* is the frequency of light, *e* is the electronic charge, 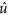 is the vector defining the polarization of the incident electric field, and 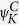 and 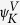
are the conduction and valence band wave functions at k, respectively. The real part *ε*_1_(*ω*) of the dielectric function can be derived from the imaginary part by the Kramers-Kronig relations. The other optical properties, such as the refractive index, absorption spectrum, loss-function, reflectivity and photoconductivity are derived from *ε*_1_(*ω*) and *ε*_2_(*ω*)^30^. In metal and metal-like systems there are intraband contributions from the conduction electrons mainly in the low-energy infrared part of the spectra. So an empirical Drude term with plasma frequency 3 eV and damping 0.05 eV is used for the dielectric function in our calculation. From the analysis of the band structure and DOS, MoAlB behaves as a metallic compound, so 0.5 eV Gaussian smearing is used in all calculations. Figure 6 presents the dielectric function, refractive index for incident photon energies up to 45 eV.

The dielectric function is the most general property of a material and can characterize how a material responds to the incident electromagnetic wave of light. Figure 6(a) presents the real and imaginary parts of the dielectric function. The peak of *ε*_2_(*ω*) is related to the electron excitation. The large negative value of *ε*_1_(*ω*) shows that the MoAlB crystal has a Drude-like behavior. In Fig. 6(a), *ε*_2_(*ω*) approaches zero from above about 18 eV, which is an additional indication of metallic conductivity. In the high energy region (ultraviolet region), *ε*_1_(*ω*) is close to unity and *ε*_2_(*ω*) reaches nearly zero, which indicates that MoAlB becomes almost transparent with very little absorption. The static dielectric constant *ε*_1_(0) is about 48.3 at a photon energy of zero, which is much larger than those of BaTiO_3_ (5.12), BiInO_3_ (6.75), Ti_3_N_4_(18.31)^31^,^32^,^33^,^34^. This shows that MoAlB is a promising dielectric material.

The refractive index is another important property of optical materials and its real part *n* indicates the phase velocity, while its imaginary part *k* is often called the extinction coefficient and indicates the amount of absorption loss when the electromagnetic wave propagates through the material. For MoAlB, the static refractive index *n*(0) is about 20 from Fig. 6(b). *n* decreases drastically and then increases to its highest peak at around 35 eV. The extinction coefficients *k* first increases drastically to 3.2 at about 5.5 eV and then decreases to the minimum value at about 20 eV. In the photon energy range from 4.8 eV to 20.4 eV, the extinction coefficient *k* is larger than the refractive index *n*, which means that light cannot propagate in this region.

**Figure 7:**
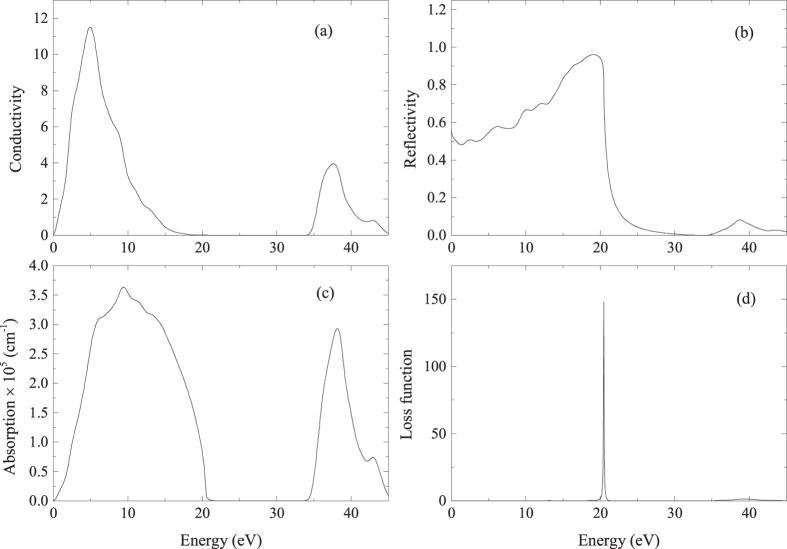
Optical properties of MoAlB. (**a**) Conductivity; (**b**) Reflectivity; (**c**) Absorption spectrum; (**d**) Loss function.

Figure 7 presents the conductivity, absorption, energy-loss function and reflectivity for incident photon energies up to 45 eV. Optical conductivity is a good gauge of photoconductivity that could shed light on the electrical conductivity of the material^35^. Since MoAlB is metallic with no band gap, Fig. 7(a) presents the photoconductivity starting with zero photon energy. The optical conductivity σ shows a sharp increase to reach the maximum value of ~11.50 in the energy range from 4.3 to 5.0 eV in the ultraviolet region and then decreases to the minimum, then increases to reach the second peak from 37.2 to 37.9 eV. Obviously, MoAlB should be more conductive when the incident photon energy ranges from 4.3 eV to 5.0 eV.

The reflectivity is the ratio of the energy of a wave reflected from a surface to the energy of the wave incident on the surface. Figure 7(b) presented the reflectivity spectra as a function of photon energy. The reflectivity of MoAlB starts from about 0.9, increases to reach the maximum value of about 0.95 at the photon energy of about 19 eV in the ultra-violet region, then decreases drastically to reach the minimum. This indicates that MoAlB possesses high reflectivity in the energy range up to ~19 eV, and the reflectivity decreases to a very low value (high transparency) for short wavelengths.

The absorption coefficient defines the extent to which a material absorbs energy. Figure 7(c) presents the absorption spectrum of the title compound. It is noted that the absorption spectrum begins at zero photon energy because of its metallic nature and rises sharply with the highest peak of 3.63409 × 10^5^ cm^−1^ at 9.4 eV. The absorption spectrum decreases sharply from 12.6 eV to 23 eV and rises from 33.0 eV to 38.1 eV with the second peak of 2.93112 × 10^5^ cm^−1^ at 38.1 eV. So the frequency area of 0–23.0 eV is the strongest absorption zone for MoAlB.

The energy loss function can be used to describe the optical spectra and the excitations produced by swift charges in a solid can be obtained from the imaginary part of the reciprocal of the complex dielectric function. Figure 7(d) shows the energy loss spectrum of MoAlB. The highest peak of the energy loss function appears at a particular incident light frequency known as the plasma frequency ω_p_^36^ of the material. The plasma frequency of MoAlB is 20.4 eV and corresponds to the rapid decrease of reflectivity in Fig. 7(b). This shows that MoAlB will change from a metallic to a dielectric response if the incident light has a frequency greater than 20.4 eV. It is noted that the energy loss spectrum does not exhibit any distinct maxima in the range from 0 to 20 eV because of the larger ε_2_^37^.

## Conclusion

First-principles calculations have been performed to investigate the structural, electronic and optical properties of the boron-containing MAX phase MoAlB. The theoretical lattice parameters are in good agreement with the available experimental values. The electronic structure of MoAlB shows metallic behavior. The Mo-B bond is partially covalent and has an ionicity with B withdrawing charge from Mo. The phonon calculation confirms the dynamical stability and the low DOS at the Fermi energy indicates the structural stability of MoAlB. The optical properties are investigated. MoAlB should be more conductive in the energy rang of 4.3–5.0 eV and changes from a metallic to dielectric response if the incident light has a frequency greater than 20.4 eV. In addition, MoAlB possesses high reflectivity in the energy range up to ~19 eV and its plasma frequency is 20.4 eV. Light cannot propagate in the photon energy range from 4.8 eV to 20.4 eV for MoAlB.

## Methods

The space group for MoAlB is *Cmcm* space group^10^. Density functional calculation implemented in the Vienna *ab-initio* simulation package (VASP) code^38^ were performed for the energy and electronic structure of MoAlB crystal. The generalized gradient approximation (GGA) with the Perdew-Burke-Ernzerhof (PBE) functional^39^ for exchange and correlation is employed. The adopted method is all-electron projector augmented wave (PAW) method^40^ for Mo, Al, and B atoms with valence electrons of 4s^1^3d^5^, 3s^2^3p^1^ and 2s^2^2p^1^, respectively.

During geometry optimization, no symmetry was used and no constraints were applied for the unit cell and the atomic positions, and a plane-wave cutoff energy of 850 eV was used. The *k*-points sampling in the Brillouin zone was 9 × 9 × 9 based on the Monkhorst-Pack method in order to ensure the energy convergence with energy differences of less than 1 meV per atom. The band structure and electron localization function (ELF) were also calculated. To obtain the electronic density of state (DOS), the tetrahedron method with Bloch corrections was used for the Brillouin-zone integration and 11 × 11 × 11 *k*-points sampling was used. The phonon structures were determined by using a supercell approach implemented in the PHONOPY code^41^.

Elastic constants were calculated by using CASTEP (Cambridge Serial Total Energy Package) program^18^. The bulk modulus B and shear modulus G were obtained from the calculated elastic constants *C*_*ij*_. And the Vickers hardenss (*H*_*ν*_) was estimated by using the empirical mode correlating the elastic moduli and Vickers hardness.

## Additional Information

**How to cite this article:** Li, X. *et al*. First-principles study of the electronic and optical properties of a new metallic MoAlB. *Sci. Rep.* **6**, 39790; doi: 10.1038/srep39790 (2016).

**Publisher's note:** Springer Nature remains neutral with regard to jurisdictional claims in published maps and institutional affiliations.

## Figures and Tables

**Table 1:** Calculated lattice constants (a, b, c) (Å), crystal cell volume V_0_ (Å^3^), position of the atoms and elastic moduli of bulk MoAlB at P = 0 GPa and T = 0 K, together with the experimental and other theoretical data.

	MoAlB	
	Calculated value	Experimental value
Lattice constants (Å)	(3.1898, 13.9024, 3.0982)	(3.1987, 13.9218, 3.0937)^10^
		(3.21, 13.98, 3.10)^17^
V_0_ (Å^3^)	136.84	137.77^10^
Atomic positions	Mo (0, 0.41055, 0.25)	Mo (0, 0.41052, 0.25)^10^
	Al (0, 0.19853, 0.25)	Al (0, 0.19849, 0.25)^10^
	B (0, 0.03346, 0.25)	B (0, 0.03383, 0.25)^10^
B (GPa)	210.99	—
G (GPa)	114.70	—
E (GPa)	291.31	—
ν	0.27	—
H_ν_ (GPa)	12.71	13.6^21^; 10.36^17^
